# Shaping the Growth Behaviour of Biofilms Initiated from Bacterial Aggregates

**DOI:** 10.1371/journal.pone.0149683

**Published:** 2016-03-02

**Authors:** Gavin Melaugh, Jaime Hutchison, Kasper Nørskov Kragh, Yasuhiko Irie, Aled Roberts, Thomas Bjarnsholt, Stephen P. Diggle, Vernita D. Gordon, Rosalind J. Allen

**Affiliations:** 1 School of Physics and Astronomy, University of Edinburgh, James Clerk Maxwell Building, Peter Guthrie Tait Road, Edinburgh, EH9 3FD, United Kingdom; 2 Center for Nonlinear Dynamics and Department of Physics, The University of Texas at Austin, Austin, Texas 78712-1199, United States of America; 3 Department of International Health, Immunology and Microbiology, Faculty Of Health Sciences, University of Copenhagen, DK-2200 Copenhagen, Denmark; 4 School of Life Sciences, Centre for Biomolecular Sciences, University of Nottingham, University Park, Nottingham NG7 2RD, United Kingdom; 5 Department of Biology & Biochemistry, University of Bath, Claverton Down, Bath BA2 7AY, United Kingdom; 6 Department for Clinical Microbiology, University of Copenhagen, DK-2100 Copenhagen, Denmark; Ghent University, BELGIUM

## Abstract

Bacterial biofilms are usually assumed to originate from individual cells deposited on a surface. However, many biofilm-forming bacteria tend to aggregate in the planktonic phase so that it is possible that many natural and infectious biofilms originate wholly or partially from pre-formed cell aggregates. Here, we use agent-based computer simulations to investigate the role of pre-formed aggregates in biofilm development. Focusing on the initial shape the aggregate forms on the surface, we find that the degree of spreading of an aggregate on a surface can play an important role in determining its eventual fate during biofilm development. Specifically, initially spread aggregates perform better when competition with surrounding unaggregated bacterial cells is low, while initially rounded aggregates perform better when competition with surrounding unaggregated cells is high. These contrasting outcomes are governed by a trade-off between aggregate surface area and height. Our results provide new insight into biofilm formation and development, and reveal new factors that may be at play in the social evolution of biofilm communities.

## Introduction

Surface-attached communities known as biofilms are believed to be the predominant mode of existence for bacteria in many environmental settings [1]. Understanding how biofilms establish and grow is also clinically important given their ubiquity in medical implant infections [2], chronic wounds [3], and in the respiratory tracts of cystic fibrosis patients [4]. In the clinical context, biofilm communities often show enhanced virulence [5], resistance to antibiotics [6], and resistance to the host immune system [7]. These features may be associated with the spatial structure of the biofilm, which not only affects material transport, e.g., penetration of nutrients/antibiotics, but is also associated with differences in metabolism and gene expression among cells within the community [8, 9].

In the canonical picture of biofilm development, individual cells land on a surface, attach and proliferate to form first micro-colonies and later 3-dimensional structures [10]. However, bacteria are also known to form dense aggregated clumps when they are grown in liquid (planktonic phase) [11–13]. Moreover, cells often disperse from existing biofilms as clumps of aggregated cells. Thus it is very likely that when a biofilm forms, some cells may arrive on the surface already in an aggregated state. In support of this view, evidence exists for the seeding of infections by pathogenic bacteria already in an aggregated state [14, 15], and bacterial aggregates are abundant in cystic fibrosis [4, 5] and tuberculosis [16] infections.

Having arrived on the surface, e.g., a plant leaf [17], a surgical implant [2] or an industrial component [18], it is to be expected that cells within a bacterial aggregate will have to compete during biofilm development, both with other aggregates and with initially non-aggregated cells, to which they may or may not be genetically related.

We take a first step towards understanding the role of pre-formed aggregates in biofilm development by investigating this competitive process, using agent-based simulations. Such simulations, in which the spatial structure of a biofilm emerges from local interactions between individual cells, have become a staple tool for investigating biofilm structure and dynamics [19–21], as well as social evolutionary aspects of biofilm development [22, 23]. Using this approach, we determine how a pre-existing aggregate of bacteria impacts the spatial structure of a biofilm, both in the presence and absence of competing unaggregated bacterial cells.

Our main focus here is on the role of the initial shape of the aggregate. It is well known that bacterial interactions with a surface depend on features such as extra-cellular polymeric substances (EPS), presence of cell surface appendages (such as pili), and cell surface charge, which are species- and strain-dependent [24]. Moreover, soft-matter science has established that the nature of material-surface interactions can drastically affect the shape of fluid or semi-fluid droplets on surfaces [25]. It is therefore reasonable to suppose that in some circumstances, bacterial aggregates will spread out in contact with a surface, while in other scenarios, aggregates will adopt a more compact configuration. Here we investigate the biological consequences of aggregate shape in the seeding of biofilm growth.

Simulating the development of biofilms initiated from initially spread or rounded aggregates, we find that the initial configuration of a bacterial aggregate on a surface is crucial in determining its eventual fate within the biofilm. In the absence of competitor cells on the surface, aggregates that maximise the extent to which they initially spread on the surface perform better than rounded ones because their initial access to nutrients (in the surrounding media) is greater. However when faced with strong competition from neighbouring unaggregated cells, initially rounded aggregates perform better over long durations, despite the fact that the rounded aggregate shape has a smaller surface area and hence a reduced exposure to nutrients. Importantly, we show that in an initially rounded aggregate, cells at the top of the aggregate proliferate at the expense of cells in the aggregate centre. This has interesting possible consequences for social evolution given that cooperation within clumps of aggregated cells has been suggested to be a stepping stone in the evolution of multicellularity [26, 27].

Our study highlights the effects of nutrient gradients and bacterial aggregate shape on long-term biofilm development. Our work reveals that these factors alone can produce a trade-off between nutrient access and competition, with the balance between these factors depending sensitively on aggregate shape. While the link between biofilm spatial structure and nutrient access has been highlighted in many other studies [8, 23, 28–30], our work is the first to focus on the role of pre-formed aggregates in this context. Our study should help to decipher the role of pre-formed aggregates in biofilm infections. More generally, our findings emphasise the need to consider pre-formed aggregates in our current understanding of biofilm development.

## Methods

**Fig 1:**
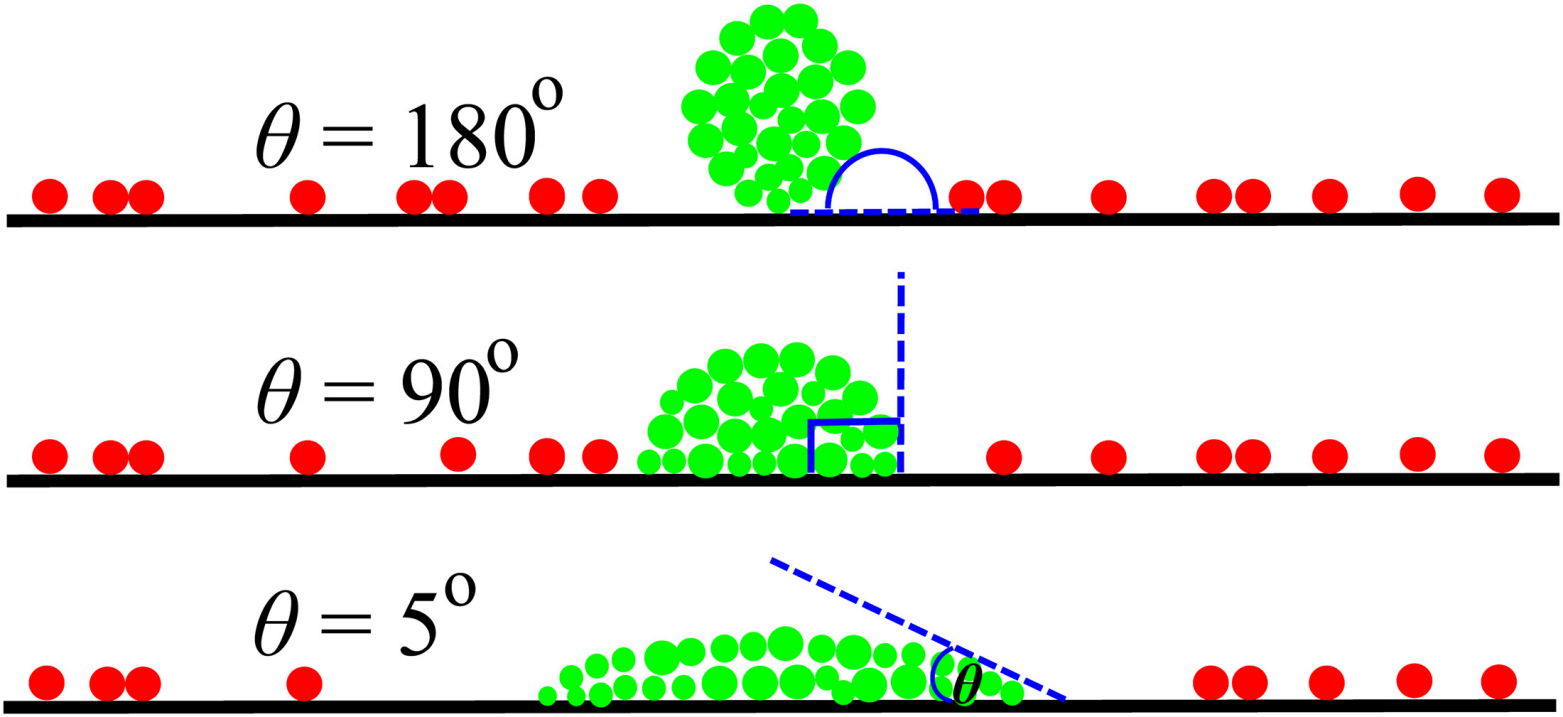
Our simulation set-up. Schematic representation of bacterial aggregates (green) which are initially spread on a surface to varying extents. The schematic also shows surrounding, competing, unaggregated cells (red). *θ* is the angle where the aggregate-medium (nutrient) interface meets the solid surface (see Section A in S1 File). Aggregates were generated from pre-formed biofilms by extracting cells whose coordinates lay within circular geometries (defined by *θ*) of varying size (see Section A in S1 File). Top- Rounded aggregate, *θ* = 180°; Middle- Semi-spread aggregate, *θ* = 90°; Bottom- Spread aggregate with *θ* = 5°. Note that the size of the aggregates (in terms of number of bacteria) is approximately equal.

In this study, we used agent-based computer simulations to model the growth of a biofilm on a surface, starting from initial configurations of bacterial cells like those shown in Fig 1. In our simulations, an initial aggregate of cells (shown in green in Fig 1), adopting a particular shape, seeds an inert surface, and may compete with surrounding unaggregated cells (red in Fig 1). Note that the red and green bacterial cells differ only in the manner in which they are initially arranged on the surface. At the start of our simulations, the “red” bacteria were placed at random locations uniformly distributed across those parts of the surface not occupied by the aggregate (see Sections A-E in S1 File). To vary the extent of competition between the aggregated and unaggregated cells, we varied the initial cell density (number of cells per unit length of surface) of the unaggregated “red” cells (see Sections A-E in S1 File). As a control, we also ran simulations in which the aggregate grew in the absence of the unaggregated cells.

The focus of this work is on the shape of the initial cell aggregate. Fig 1 illustrates three different scenarios, in which the cell aggregate adopts a compact rounded shape (top), spreads out on the surface (bottom), or adopts an intermediate shape (middle). In each scenario, the dimensions of the aggregate are adjusted so that the number of cells within the aggregate remains the same (see Sections A and B in S1 File).

### Aggregate shape characteristation

To characterise quantitatively the aggregate shapes in the different scenarios shown in Fig 1, we defined the “aggregate-surface angle” *θ*, which is the angle that the initial aggregate makes with the flat surface. A small value of *θ* (*θ* → 0°) describes an initial aggregate configuration that is spread on the surface, whereas a large value of *θ* (*θ* → 180°) describes an aggregate that is rounded. Given that the total number of cells in the initial aggregate is fixed, *θ* also encapsulates an interplay between the initial surface coverage of the aggregate and its initial height; with increasing *θ*, the surface coverage decreases whereas height increases. In soft matter science, an analogous parameter is often used to describe wetting interactions between liquid droplets and surfaces [25]. A similar approach has recently been applied to the surface-spreading behaviour of eukaryotic cell aggregates [31]. From a phenotypic perspective, *θ* is related to the nature of the interactions between cells in the aggregate and between cells and the surface, and thus could be tunable by biological regulatory processes, or by evolution.

As *θ* → 0° the aggregate is no longer defined (since it would become an infinitely thin line along the surface) thus it is impossible to reach a value of *θ* = 0. Therefore to explore the effect of the initial aggregate configuration on the surface, we performed simulations for a range of *θ* values between 5° (spread) and 180° (rounded).

The aggregate configurations that we used to initiate our simulations were generated by “transplantation” of circular segments from simulation snapshots of pre-grown biofilms (see Sections A and B in S1 File). This procedure proved preferable to other initialisation methods as it ensures no overlap between individual bacteria and enables the generation of different aggregate shapes of the same number density (∼100 cells per unit area). By varying the radius of the circular segment we were able to ensure that each aggregate contained ∼100 cells that were initially spread on the surface to different extents. To ensure statistical accuracy, four different configurations were generated for each aggregate shape (see Section E in S1 File), defined by its value of *θ*, and for each of these configurations, five simulations were performed using different seeds for the random number generator that governs any stochasticity within the simulations [32]. Changing the random number seed affects, amongst other things, the order in which individual bacteria grow and divide, and also changes the locations of the unaggregated cells on the surface surrounding the aggregate. A total of twenty simulations were therefore performed for each value of *θ*, enabling us to sample both variation in the configuration and the ordering of cell updates (Section E in S1 File).

In common with many other biofilm simulation studies [19, 22, 23, 33], our simulations were performed in two dimensions for the purposes of computational efficiency. We have verified, however, that our key findings are reproduced when we use 3D simulations (see Sections C and J in S1 File).

### Simulation implementation

**Fig 2:**
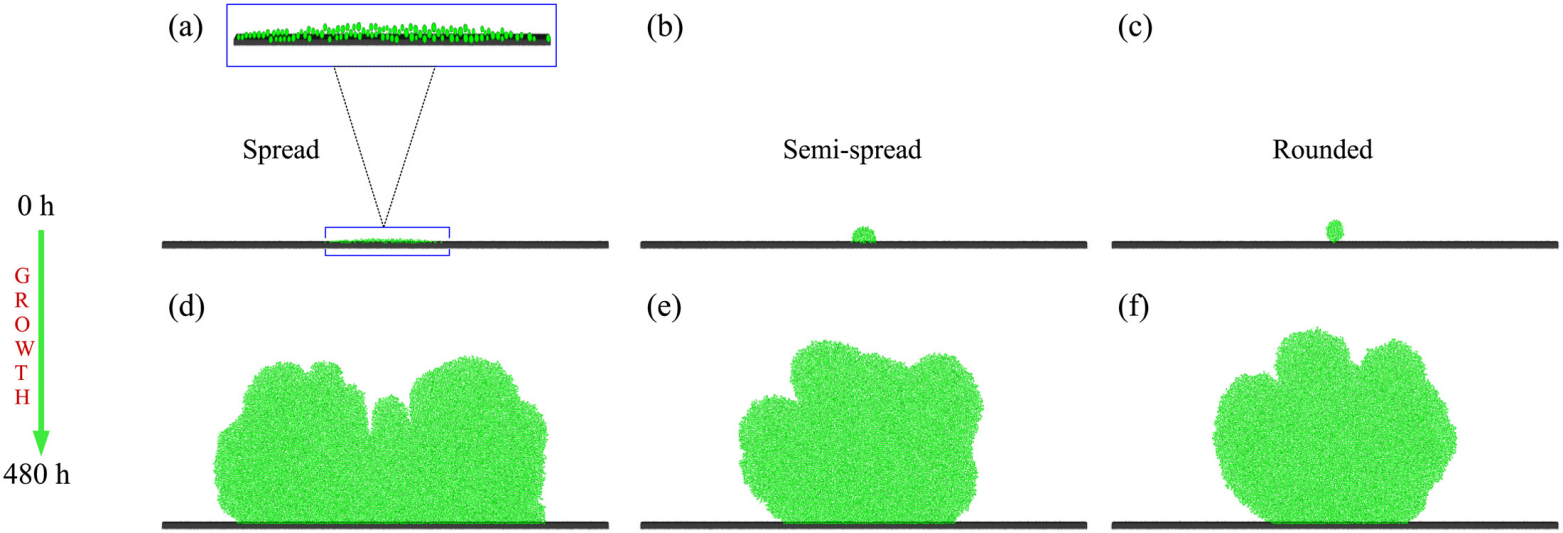
Initial aggregate arrangement affects biofilm morphology. Simulation snapshots of three bacterial aggregates initially arranged on the surface and the biofilms they form after 480 h: (a) Spread, 0 h. A zoomed in image is also shown to make the shape of the aggregate easier to resolve; (b) Semi-spread, 0 h; (c) Rounded, 0 h; (d) Spread, 480 h; (e) Semi-spread, 480 h; (f) Rounded, 480 h.

We used the agent-based microbial simulation package iDynoMiCs [32] to model biofilm growth, starting from configurations such as those shown in Fig 2. In these simulations, individual bacterial cells are represented as spherical agents, which grow and proliferate conditional on the local nutrient concentration, and “shove” each other apart to relieve local stresses within the biofilm. The order in which cells are selected to grow and divide is random and uniform during each global time-step of the simulation, as is the direction of cell division, i.e., upon division a random orientation vector, defined by an angle selected from a uniform distribution, is used to position the daughter cell away from the mother cell. A “shoving” algorithm then corrects for any overlap that results with other cells. In our simulations, the initial distribution of surrounding competitor cells on the surface is also random and uniform. For more information on the stochastic aspects of iDynoMiCs, we refer the reader to [32]. The simulations use a spatial grid to track the local nutrient concentration field. Nutrient is assumed to diffuse towards the biofilm from above, with the concentration being fixed to a bulk value in a layer far from the biofilm. Within the biofilm itself, nutrient diffusion is hindered relative to the region outside the biofilm. Nutrient consumption by the bacterial cells leads to local gradients, which can have a strong impact on the structural features of the growing biofilm [8, 23, 28–30]. Periodic boundary conditions are imposed on both the nutrient concentration field and the particle coordinates in the horizontal direction.

From a mathematical perspective, nutrient is represented as a concentration field, the dynamics of which are governed by the reaction-diffusion equation

$$\begin{array}{c} \frac{\partial S(x)}{\partial t}=\nabla \cdot \left(D_{S}\left(x\right)\cdot \nabla S\left(x\right)\right)+r_{S}\left(x\right), \end{array}$$ (1)

where *S*(x) is the space (x)-dependent nutrient concentration, *D_S_*(x) is the diffusion coefficient of the nutrient, and *r_S_*(x) is the consumption rate of the nutrient by the bacteria. The rate of nutrient consumption, *r_S_*(x), is related to the growth rate of the bacteria, *dX*/*dt*, via

$$\begin{array}{c} r_{S}\left(x\right)=\frac{dS}{dt}=-\frac{1}{Y_{x/s}}\;\frac{dX}{dt}, \end{array}$$ (2)

where *X*(x) is the local biomass density, and *Y*_*x*/*s*_ is a yield coefficient that describes the amount of nutrient required to produce one unit of biomass *X*.

The growth rate of each cell is governed by the well-known Monod function

$$\begin{array}{c} \frac{dX}{dt}=\mu_{max}\;\frac{S}{k_{S}+S}X, \end{array}$$ (3)

where *μ*_*max*_ is the maximum specific growth rate of the bacteria, and *k*_*S*_ is the concentration of nutrient, *S*, at which the growth rate is half maximal. The growth parameters used in our simulations were taken from empirical and simulation studies on *Pseudomonas aeruginosa*, assuming glucose to be the rate-limiting nutrient (see Table 1). Note that the growth rate parameters *Y*_*X*/*N*_, *μ*_*max*_, and *k*_*S*_ are the same for both the aggregate cells and the competitor cells (see Table 1).

**Table 1:** Input parameters for biofilm simulations.

Symbol	Description	Value	Notes/ref
*S*_*bulk*_	Bulk concentration of limiting nutrient	5.4 × 10^−3^ g L^−1^	Within range of values from [32–34]
*Y*_*x*/*s*_	Yield coefficient for Monod equation (Eq 3)	0.44	Within range of values from [33, 35, 36]
*μ*_*max*_	Maximum specific growth rate	0.35 h^−1^	Within range of values from [35, 37–39]
*k*_*s*_	Half saturation concentration of nutrient	3 × 10^−3^	Within range of values from [20, 21, 32, 35–38, 40]
*γ*	Density of biomass	200 g L^−1^	[20, 33, 35]
*D*	Diffusivity of glucose in water	5.8^−5^ m^2^ day^−1^	[41]
*L*_*x*_	Dimension of system in horizontal direction	1032 *μ*m	Ensures aggregates do not interact periodically
*L*_*y*_	Dimension of system in vertical direction	1032 *μ*m	Corresponds to the horizontal length
*L*_*dbl*_	Thickness of diffusion boundary layer	80 *μ*m	Within range of values from [20, 21, 42]

From a practical point of view, in iDynoMiCs the nutrient concentration fields are assumed to be in pseudo steady-state with respect to biomass growth and therefore the time dependence is removed from Eq 1

$$\begin{array}{c} 0=\nabla \cdot \left(D_{S}\left(x\right)\cdot \nabla S\left(x\right)\right)+r_{S}\left(x\right). \end{array}$$ (4)

This equation is solved numerically for every global update of the bacterial population. In our simulations, we used a bulk nutrient concentration of 5.4 × 10^−3^ g L^−1^ (see Section F in S1 File) comparable with previous work [32–34]. Each of our simulations was run for a total time of 480 h, in order to explore the long-term growth dynamics (see Results and Section G in S1 File). Our complete parameter set is listed in Table 1. Using the parameters in Table 1, our simulations produce spatially structured biofilms 200–300 *μ*m in height after a simulation time of 480 h (see Section F in S1 File).

## Results

### Initial aggregate shape determines growth dynamics

**Fig 3:**
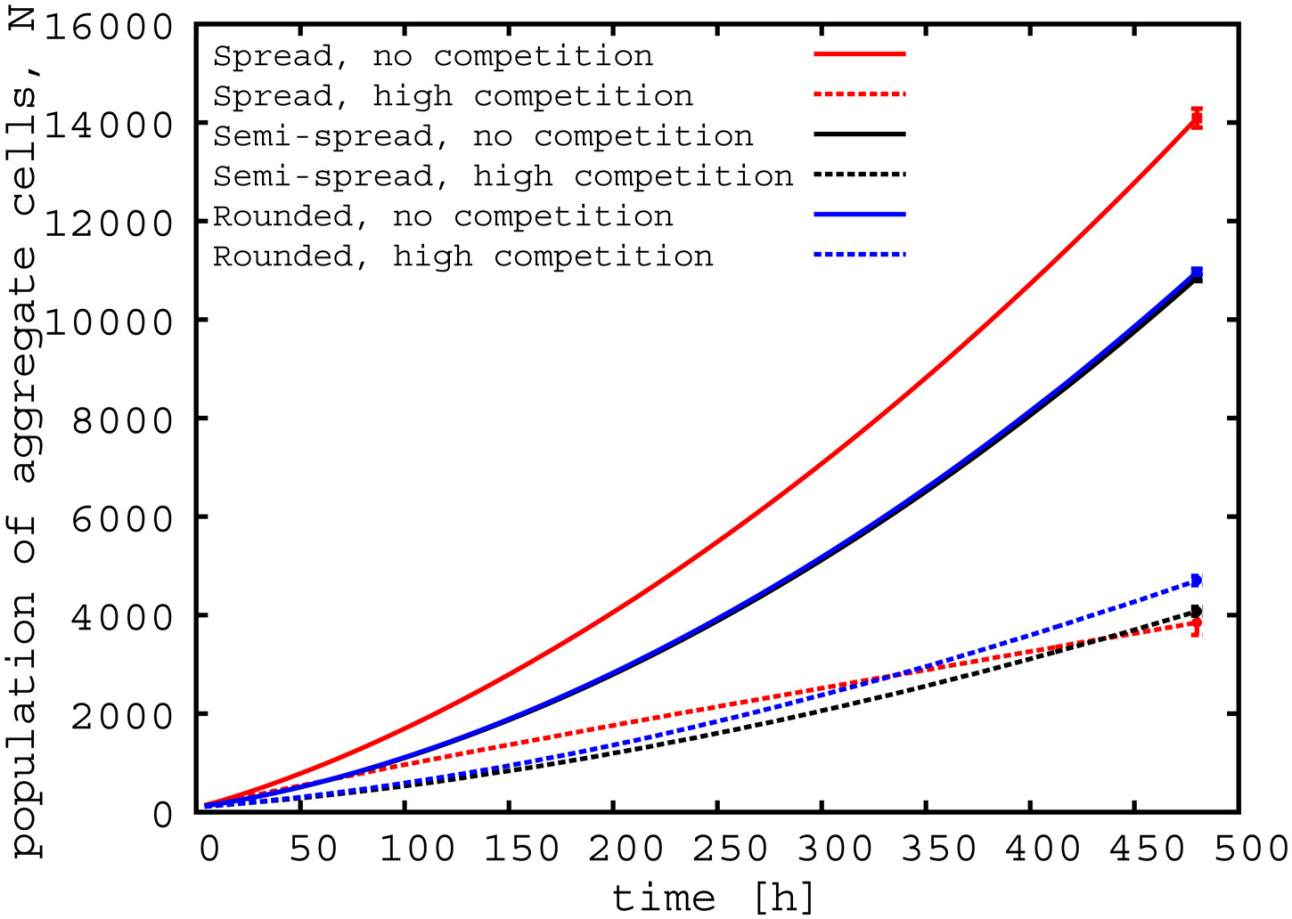
Aggregate shape governs growth dynamics. Growth of the spread (*θ* = 5^*o*^), semi-spread (*θ* = 90°), and rounded aggregate (*θ* = 180°) populations over the course of our simulations in the absence (*ρ* = 0 cell *μ*m^−1^) and presence (*ρ* = 0.5 cell *μ*m^−1^) of competition. For clarity the error bars, representing the standard deviations, are only shown for the final data points. The standard deviations at these points are maximal.

To assess the growth dynamics of pre-formed aggregates in a biofilm, we tracked the number of progeny cells, *N*, produced by aggregates of different shape (Fig 3). We first investigated three different aggregate shapes, characterised by the angle *θ* (See Methods). The three angles, *θ* = 5°, 90°, 180°, describe pre-formed aggregates that are initially arranged on the surface in either a spread, intermediately spread, or rounded manner. To investigate the effect of the surrounding unaggregated competitor cells (red cells in Fig 1), we varied the density, *ρ*, of these cells between two extreme regimes of competition reported in this study: *ρ* = 0 cell *μ*m^−1^, no competition; and *ρ* = 0.5 cell *μ*m^−1^, high competition.

Fig 3 shows that the final population of cells originating from the aggregate depends on both the presence of competition with surrounding cells on the surface and the initial shape of the aggregate (two way ANOVA with replication; competition, degrees of freedom (df) = 1, P < 0.001; shape, df = 2, P < 0.001; interaction, df = 2, P < 0.001). Not surprisingly, aggregates grow better in the absence of surrounding cells on the surface regardless of their initial shape, i.e., for *ρ* = 0 cell *μ*m^−1^. In this “non-competitive regime”, the initially spread aggregate produces more progeny than the more rounded aggregate (unpaired two tailed T-test assuming unequal variances, df = 22, P < 0.001). This is evident in Fig 2, which shows representative initial aggregate configurations and the structures of the biofilms which they form after 480 hours.

Competition from unaggregated competitor cells on the surfaces leads to more complex behaviour. Fig 3 shows that, in the presence of strong competition, the spread aggregate produces more progeny over short times than the rounded aggregate. However, over longer times, the size of the population arising from the rounded aggregate is larger (unpaired two tailed T-test assuming unequal variances, df = 24, P < 0.001). Thus the advantage of the rounded aggregate only becomes important at longer times (see Section G in S1 File). For an aggregate in the presence of competing non-aggregating cells, Fig 3 points to two strategies for maximising progeny. For long-lived biofilms, progeny can be maximised by the aggregate adopting a rounded configuration (see also Section G in S1 File), whereas if the biofilm is short-lived then it may instead be optimal for the aggregate to spread on the surface.

### Initial aggregate shape affects long-term biofilm structure

In our simulations the initial shape of the aggregate influences the long-term structure of the biofilm. Fig 2 shows typical biofilm structures formed after 480 h of growth, starting from aggregates that were initially spread on the surface (*θ* = 5°, left), rounded (*θ* = 180°, right) or partially spread (*θ* = 90°, centre), in the absence of competition from surrounding cells. It is clear that the spread aggregate covers much more of the surface during growth than its more rounded counterparts.

**Fig 4:**
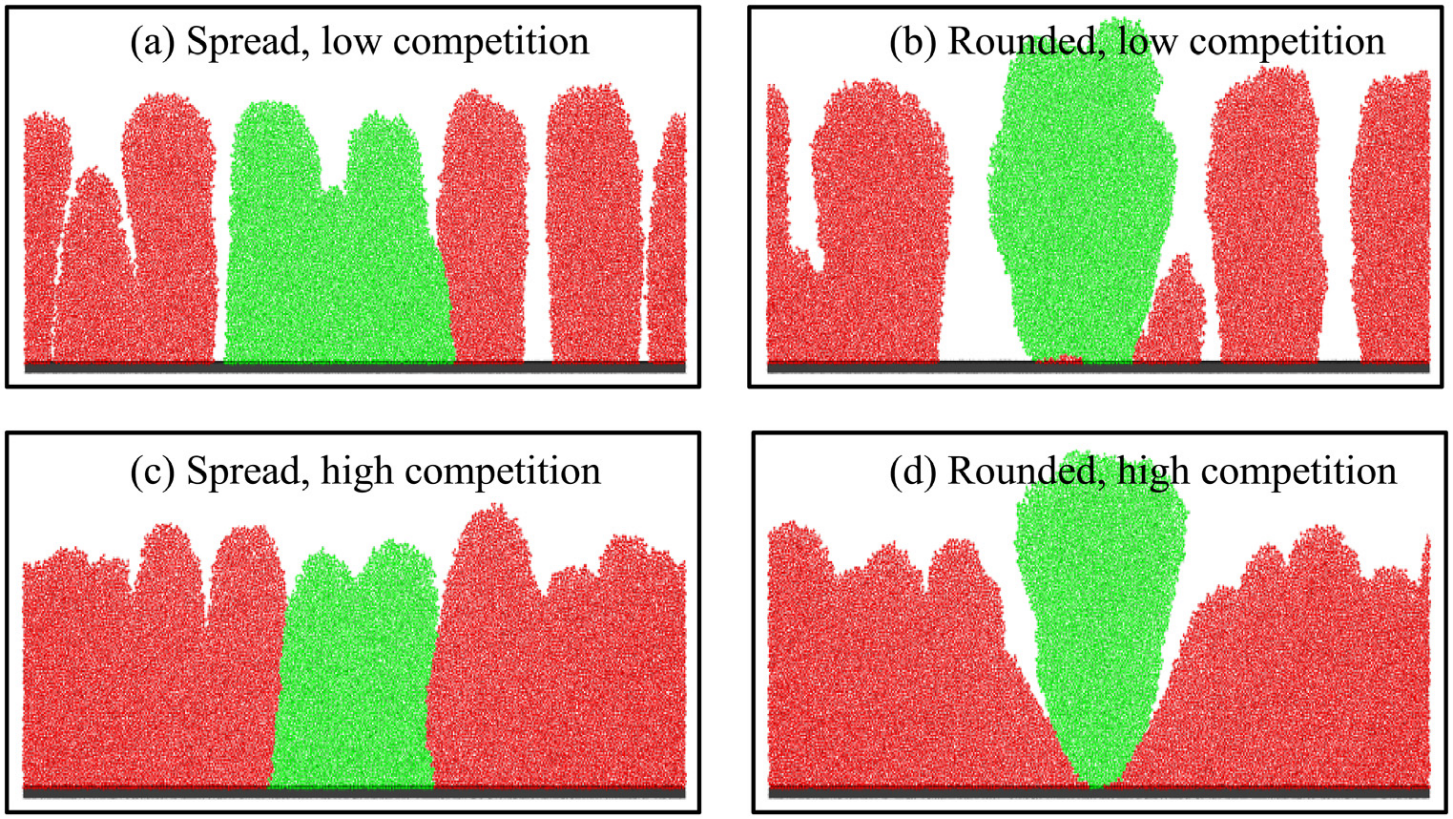
Aggregate shape and neighbouring strain density affect biofilm morphology. Simulation snapshots of biofilms seeded from spread and rounded aggregates after 480 h growth in the presence of a low and high density inoculum of the competing strain: (a) *θ* = 5°, *ρ* = 0.01 cell *μ*m^−1^; (b) *θ* = 180°, *ρ* = 0.01 cell *μ*m^−1^; (c) *θ* = 5°, *ρ* = 0.5 cell *μ*m^−1^; (d) *θ* = 180°, *ρ* = 0.5 cell *μ*m^−1^.

In the presence of competition (Fig 4), we observe a marked difference in the structure of the biofilms that originate from spread aggregates (left panels) and from rounded aggregates (right panels). For the spread aggregate (a and c), the green section of biofilm that originates from the aggregate is structurally indistinguishable to that of the surrounding red biofilm that originated from the competing, unaggregated cells. In contrast, for the rounded aggregate (b and d), cells originating from the aggregate form a distinct “clump”, which is taller than the surrounding biofilm. When the density of competing (red) cells is high (Fig 4(d)), there is a cell-free gap around the growing clump that appears to be a result of nutrient depletion.

It is clear from the biofilm structures shown in Figs 2 and 4 that, even at very long times, the spatial structure of a biofilm can be affected by the initial spatial configuration of its founder cells (see also Section F in S1 File). While it might seem remarkable that apparently small changes in initial configuration can have dramatic effects on biofilm structure even after many cell generations, this effect is in fact well known in a different context. For initially flat biofilms, Dockery and Klapper showed theoretically that small inhomogeneities in initial configuration may be magnified into large “fingers” over the course of biofilm development [30]. This phenomenon is shown as a fingering instability and arises from the fact that an emerging protrusion (or finger) is elevated above, and thus depletes nutrients from the surrounding biofilm. This leads to positive feedback, in which the enhanced growth of the cells at the top of the instability is to the detriment of the surrounding cells below [43].

While Dockery and Klapper assumed that structural inhomogeneities would arise spontaneously during biofilm growth, in our simulations such inhomogeneities are effectively created by the presence of the initial aggregates. The introduction of the rounded aggregate amongst the lawn of unaggregated cells on the surface at high competition leads to an instability in the biofilm structure that propagates as the biofilm develops.

### Nutrient gradients are important determinants of aggregate fate

**Fig 5:**
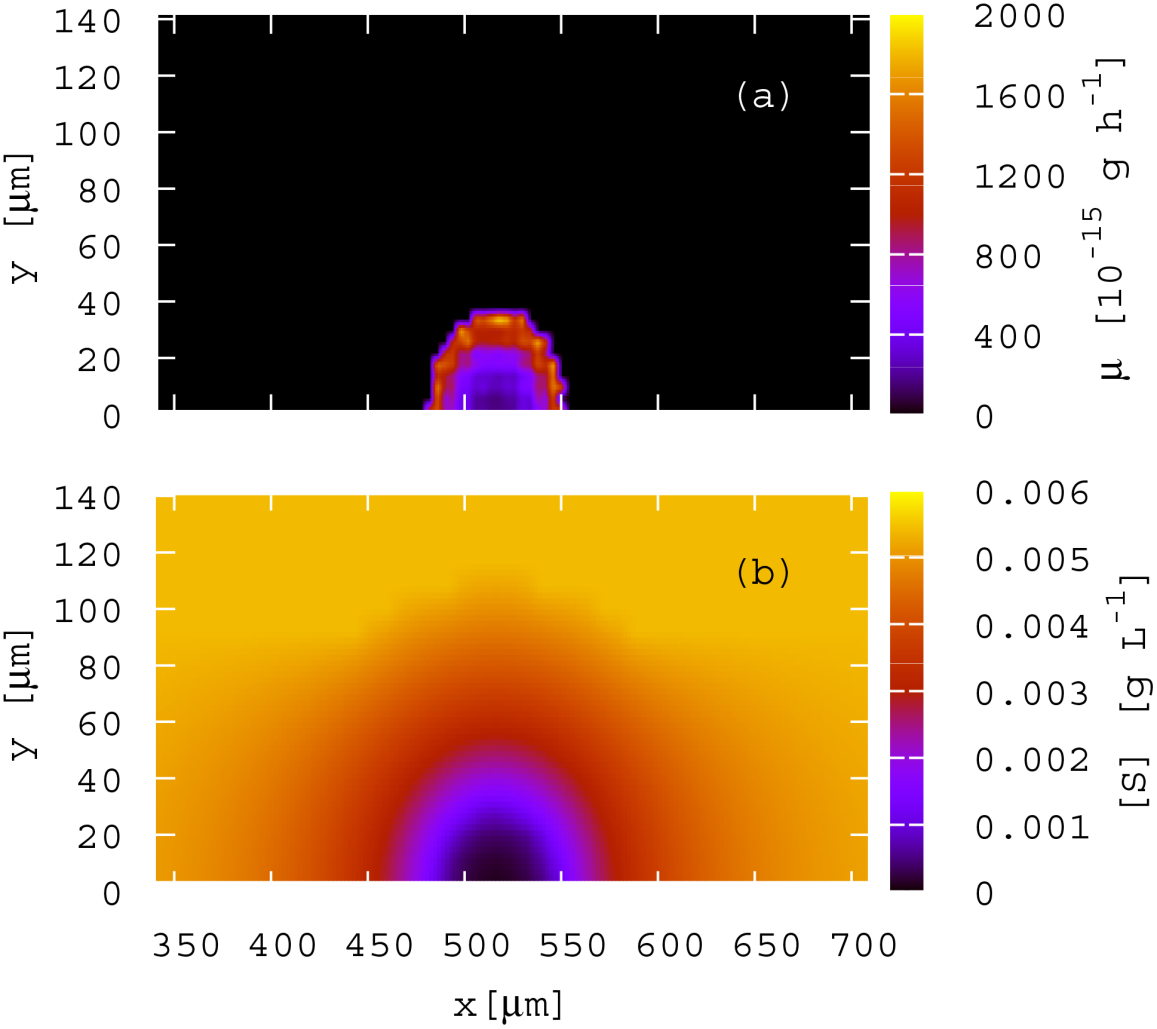
Cells on the outside of the aggregates grow faster because they have greater access to nutrients. (a) Cell growth rate (*μ*) distribution of the biofilm formed from the semi-spread aggregate in the absence of competition after 4h. (b) Corresponding nutrient concentration field, [*S*].

It is well known that growth rate heterogeneities, resulting from nutrient concentration gradients, emerge during biofilm growth [44]. With this in mind, we tracked the growth rates of individual cells as a function of their position within the growing biofilm. Even in the very early stages of biofilm growth, we see heterogeneity in growth rates which emerge from (and influence) spatial gradients in nutrient concentration. Fig 5 illustrates this for a semi-spread aggregate (*θ* = 90°), after 4 h of growth, in the absence of competition. As expected, the cell growth rate is highly heterogeneous across the biofilm, Fig 5(a), with cells on the outside growing faster than those on the inside because they have better access to nutrients (Fig 5(b)).

**Fig 6:**
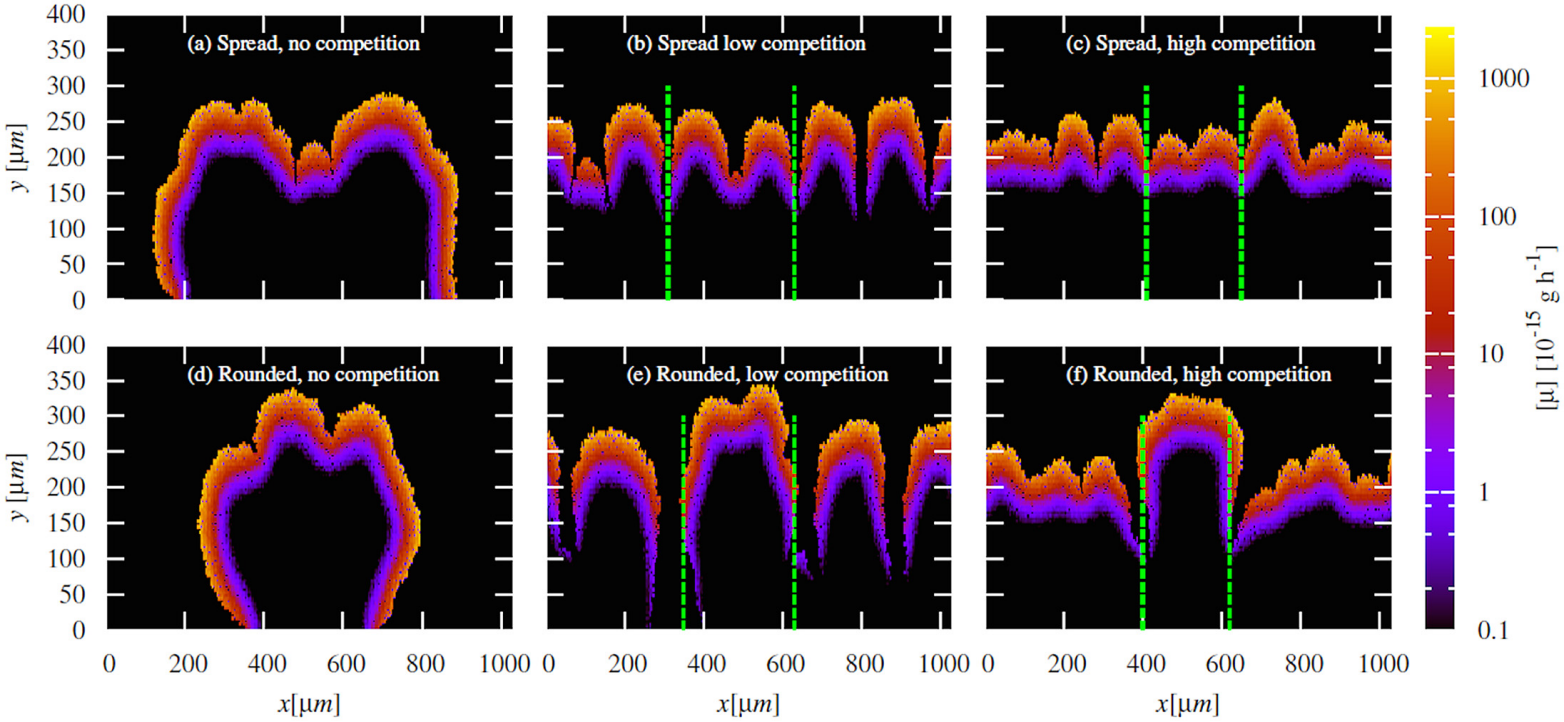
Gradients in individual cell growth rates emerge in our simulated biofilms during growth. Cell growth rate distributions for the spread and rounded aggregates after 480 h of growth: (a) *θ* = 5°, *ρ* = 0.0 cell *μ*m^−1^; (b) *θ* = 5°, *ρ* = 0.01 cell *μ*m^−1^; (c) *θ* = 5°, *ρ* = 0.5 cell *μ*m^−1^; (d) *θ* = 180°, *ρ* = 0.0 cell *μ*m^−1^; (e) *θ* = 180°, *ρ* = 0.01 cell *μ*m^−1^; (f) *θ* = 180°, *ρ* = 0.5 cell *μ*m^−1^. These distributions correspond to the configurations in Figs 2 and 4. Note that the gradient in cell growth rate is so large that a log scale is used for visualisation purposes. The green dashed lines represents an approximate boundary between the aggregate cells and the surrounding competing strain.

The growth rate heterogeneities shown in Fig 5(a) are amplified in the later stages of biofilm growth. Fig 6 shows the spatial distribution of cell growth rates for biofilms arising from spread and rounded aggregates after 480 h, in the presence and absence of competitor cells, for the same simulations as shown in Figs 2 and 4. In all cases we observe, as in previous work [45], two distinct regions of growth activity within the developing biofilms: an outer layer of metabolically active cells and an interior region of inactive cells. These distinct regions arise because consumption by cells in the outer layer deprives cells in the inner layer of nutrients [45]. We also observe a large gradient in individual cell growth rate within the growing layer itself (note the logarithmic scale in Fig 6). The dynamics of the metabolically active layer determine the overall growth behaviour and structure of the biofilm. In Section I of S1 File we show that the active layer of the rounded aggregate, unlike the spread aggregate, continues to expand in the presence of competition; explaining why its total population becomes larger than that of the spread at longer times in Fig 3, and why its structures tend to fan outwards at the top (Fig 6(f)).

Although it is well documented that nutrient gradients arising during biofilm growth play an essential role in biofilm formation [8, 23, 29], so far few studies have investigated the effect of pre-formed bacterial clumps in this process; in particular how the initial arrangement of cells within a clump affects biofilm structure and development. Figs 5 and 6 show that the initial arrangement of cells on the surface can determine the shape and structure of a growing biofilm because small initial differences in nutrient gradients become amplified as the biofilm develops.

### Competition for nutrient favours rounded aggregates

**Fig 7:**
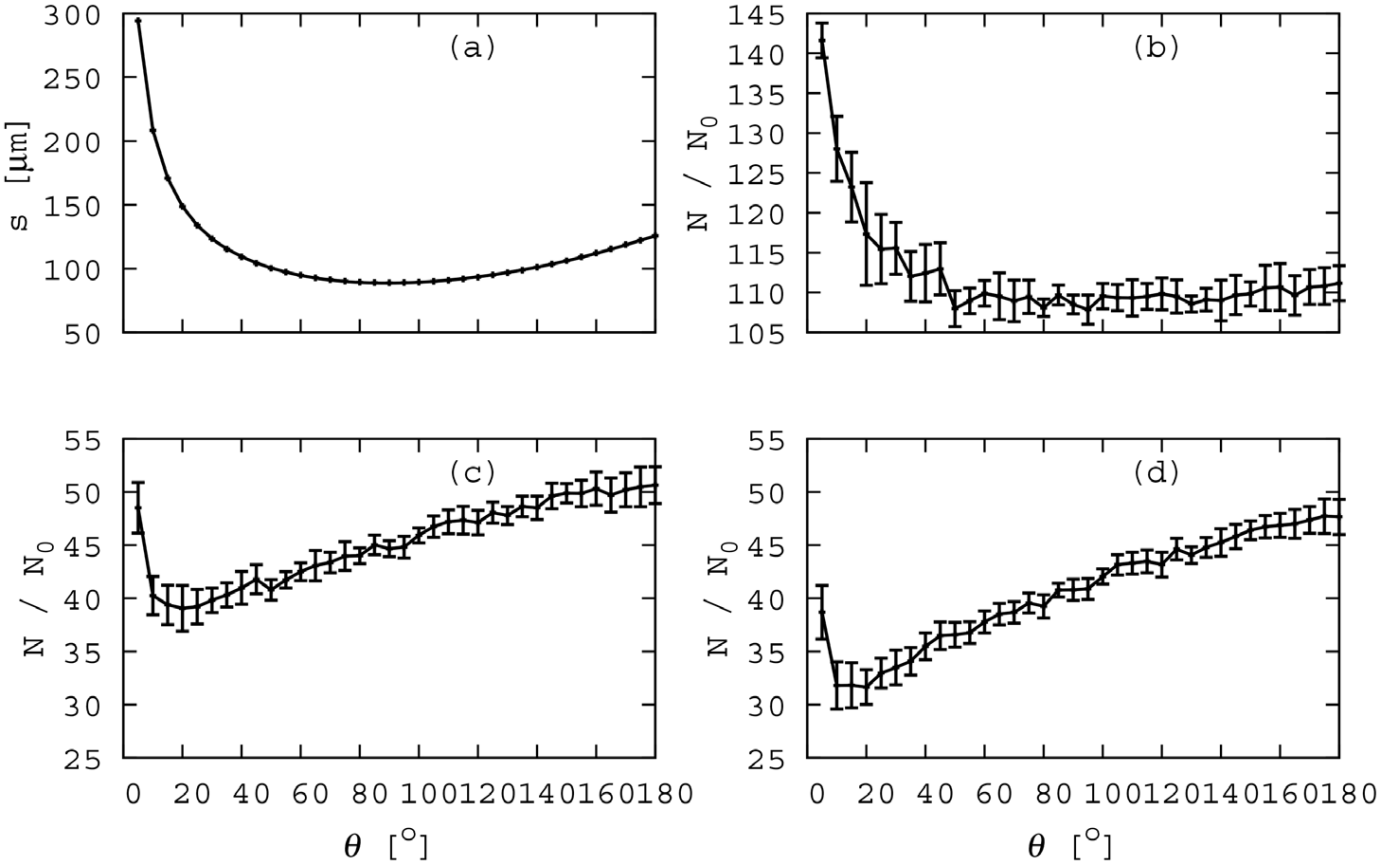
Success of aggregates depends on shape and competition. (a) aggregate-medium interface length, s, as a function of *θ*. (b-d) Average number of progeny, *N*/*N*_0_, of aggregates defined by their surface-aggregate angle *θ*, the functional from of which changes with increasing density of competitor cells: (b) *ρ* = 0 *μ*m cell^−1^; (c) *ρ* = 0.145 *μ*m cell^−1^; (d) *ρ* = 0.5 *μ*m cell^−1^. Vertical bars represent the standard deviation from 20 data points.

Next we investigated how the fate of an aggregate, as measured by the average number of progeny of one of its cells, varies with aggregate shape. To this end, we computed the number of progeny cells, *N*, arising from the aggregate after a period of biofilm growth, relative to the initial number of cells in the aggregate, *N*_0_, for a range of aggregate shapes, determined by *θ*, at varying levels of competition. For this analysis, we carried out long simulations (480 h of biofilm development), so that the ratio *N*/*N*_0_ reflects the long-time fate of the progeny of cells within the aggregate (see Section G of S1 File). Fig 7(b) shows *N*/*N*_0_ plotted as a function of the aggregate-surface angle, *θ*, in the absence of competition from surrounding unaggregated cells. It is clear that the spread aggregate produces more progeny on average than the rounded one (unpaired two tailed T-test assuming unequal variances, df = 38, P < 0.001).

In the previous section we saw that cells on the outside of the aggregate have more access to nutrients even in the very early stages of biofilm growth. Thus, we might hypothesise that the growth advantage of the spread aggregate, in the absence of competition, is related to its larger surface area. Indeed, Fig 7(a) shows that *N*/*N*_0_ correlates closely with the interfacial area (or arc length) of the initial aggregate, s(*θ*). We therefore conclude that the spread aggregate produces more progeny than the rounded one because the former has a greater surface area with the surrounding medium, providing greater exposure to nutrient in the initial stages of growth. The difference in initial structure between the spread and rounded aggregates therefore translates into significant differences in cell fate, even after many generations of biofilm growth.

**Fig 8:**
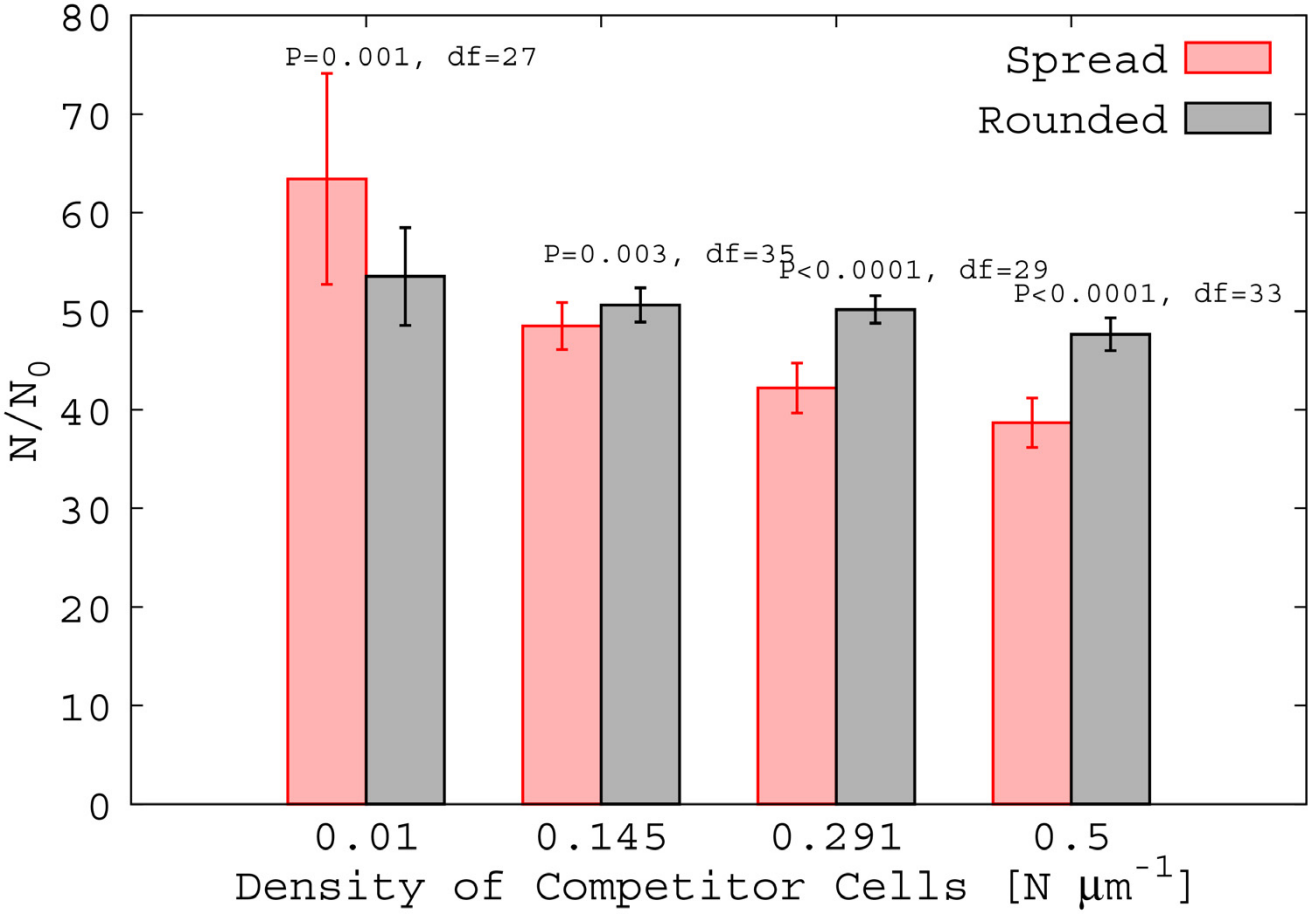
Rounded aggregate is relatively more successful with increased competition. Relative fitness as measured by *N*/*N*_0_ of rounded aggregates increases with competition. Rounded aggregates become favourable relative to spread aggregates with increasing density of competitor cells. P values and degrees of freedom computed from unpaired two tailed T-test assuming unequal variances.

As the density of competition of unaggregated cells on the surface increases, however, a very different scenario emerges. Fig 7(c) and 7(d) show that cells in the rounded aggregate (large *θ*) produce more progeny, on average, than those in the spread aggregate (unpaired two tailed T-test assuming unequal variances, (c) df = 35, P = 0.003; (d) df = 29, P < 0.001). This is more evident in Fig 8, which shows *N*/*N*_0_ for the spread and rounded aggregates as a function of the density of competitor cells. In Section H of S1 File we test how this effect depends on the nutrient concentration. Repeating our simulations for higher concentration of nutrient, we find that, for high competition, the rounded aggregate remains more successful than the spread aggregate. However, in the absence of competition there is no significant difference in outcome between spread and rounded aggregates at high nutrient concentration.

**Fig 9:**
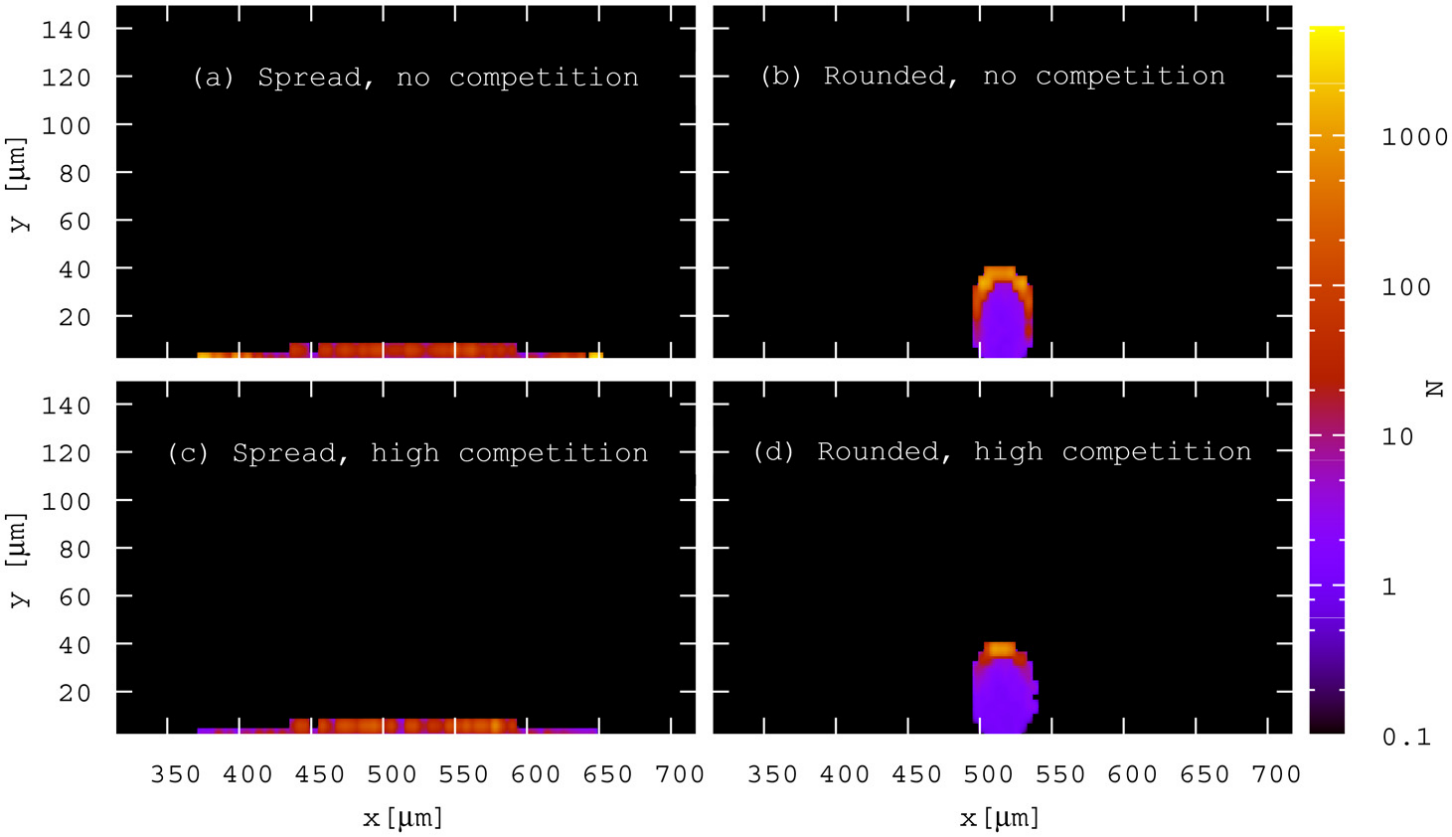
Distribution of fittest cells from the initial aggregate varies with aggregate shape. 2D histograms representing the number of progeny, *N*, produced (480 h) by individual bacteria as a function of their initial location in the spread and rounded aggregates in the absence and presence of competition: (a) *θ* = 5°, *ρ* = 0.0 cell *μ*m^−1^; (b) *θ* = 180°, *ρ* = 0.0 cell *μ*m^−1^; (c) *θ* = 5°, *ρ* = 0.5 cell *μ*m^−1^; (d) *θ* = 180°, *ρ* = 0.5 cell *μ*m^−1^. Note that these distributions were averaged over 20 trajectories for each aggregate. Note that the gradient in the number of progeny is so large that a log scale is used for visualisation purposes.

Why does competition from surrounding unaggregated cells favour rounded over spread aggregates? Close inspection of Fig 7 shows that, while the number of progeny produced by the spread aggregate decreases with increasing competition from surrounding cells (panels (c), and (d)), the number of progeny produced by the rounded aggregate remains rather constant. This finding can be understood by investigating how the fate of an individual cell within an aggregate depends on its initial spatial location. To this end, we tracked the number of progeny of each individual founder bacterium, as a function of its initial position within an aggregate. This constitutes a local, spatially-resolved version of the “fitness measure” *N*/*N*_0_. Averaging our results over 20 repeated simulations allowed us to generate a map showing the average number of progeny produced by individual cells within an aggregate, for initially spread and rounded aggregates, Fig 9.

Fig 9 shows that the initial position within an aggregate indeed has a strong effect on cell fate. In the absence of competition from surrounding unaggregated cells, the most successful cells in the spread aggregate are those at the horizontal extreme edges; in the interior region of the aggregate, cell fate is more uniform (Fig 9(a)). It seems likely that in this case, cells at the horizontal edges have an advantage because their progeny can expand in the horizontal direction, whereas the progeny of cells in the interior of the aggregate must compete with their neighbours within the aggregate for nutrients and space. The proliferation of the cells at the edges of the aggregate drives the lateral expansion of the growing biofilm which we observe in Fig 2(a) and 2(d). In contrast, for the rounded aggregate (Fig 9(b)), cell fate is overall more heterogeneous within the aggregate, with the most successful cells being located around the outside surface of the aggregate. For the rounded aggregate, it appears that height is a relevant factor as well as the proximity to the aggregate surface.

Fig 9(c) shows that, in the presence of competition, cells at the horizontal edges of the spread aggregate actually do less well than those in the centre. The decreased fitness of these cells explains the inhibited lateral expansion observed in Fig 4(a) and 4(c). For the rounded aggregate, the most successful cells in the absence of competition are those at the top of the aggregate. In the presence of competition (Fig 9(d)), these cells, which are now highly localised at the top, are elevated above the level of the competitor cells and therefore are little affected by the increased competition for nutrients. The “fitness” cost associated with its smaller surface interface is compensated in the presence of competition by its height, since its top cells remain unchallenged by competitors with respect to nutrient access.

## Discussion

Given the tendency of many bacteria to aggregate, and the frequent observation of aggregates in diverse environmental situations [5, 46, 47], it seems likely that natural biofilms are often initiated from pre-formed aggregates. Despite this, the role of pre-formed aggregates in biofilm development has, to our knowledge, not yet been addressed. In this paper, we have investigated the fate of pre-aggregated cells during biofilm formation, using individual-based simulations. Our study shows that an initial aggregate can have a significant and long-lasting effect on biofilm spatial structure, even after many generations of cell growth. Focusing on the role of aggregate shape, we find that, in the absence of competition for nutrients from surrounding cells, an aggregate that is initially spread on the surface is favoured over one that is initially rounded even over long periods of biofilm development. This is likely to be because the spread aggregate has initially a larger surface over which it can absorb nutrients, giving it an initial growth advantage that is then maintained as the biofilm grows.

Strikingly though, our results change qualitatively in the presence of competition from surrounding, unaggregated cells. When this competition is strong, although the spread aggregates still grow faster in the early stages of biofilm development, rounded aggregates become more successful (produce more progeny) as the biofilm develops over longer times. This effect appears to arise from a trade-off between height (as nutrients diffuse from above) and exposed surface area. In the absence of competition, surface area is more important than height, and the spread aggregate is favoured. However, in the presence of competition, height becomes more important, since cells at the top of the aggregate can avoid competing for nutrients with the surrounding competitors. Since the rounded aggregate is taller than the spread aggregate, it gains a “fitness” advantage under conditions of strong competition that is only realised after long times.

Bacterial biofilm formation is a complex phenomenon which involves a plethora of biological mechanisms including cell motility [48], EPS production [49, 50], metabolic and other phenotypic differentiation [9, 47, 51], and cell-cell interactions such as quorum sensing [52–54]. In our simulations, almost all of this biological complexity has been neglected; our model takes account only of nutrient gradients established by cell consumption, nutrient-dependent growth, and competition among cells for space. Nevertheless this simplistic approach produces biologically interesting, and potentially testable, predictions. In particular our simulations predict that being initially spread on a surface is a better strategy for a bacterial aggregate in the absence, but not in the presence, of competition. Understanding how further biological complexity might affect this picture would be a very interesting topic for further work. Another avenue worth investigating would be the effects of biofilm erosion and the subsequent detachment of cells. Here, our simulations have not included the effects of fluid flow, which among other effects, may flatten the biofilm by detaching protruding cells or clumps. In such cases, the initial height advantage of the rounded aggregate might be detrimental as its progeny cells are more likely to sloughed from the biofilm first. If however, it is desirable to colonise new surfaces, for instance downstream, then being sloughed off quickly might be beneficial. In reality, the effect of fluid flow on aggregated phenotypes within biofilms is much more complex because the cells within such aggregates may be more resistance to sloughing due to a high degree of cohesive interactions between the cells.

How might aggregates of different shape arise in nature? It is well known that bacterial interactions with surfaces can vary greatly depending both on the physical and chemical properties of the surface [55–57], and on bacterial phenotypes such as EPS production and the presence of surface appendages. It is therefore very likely that aggregates formed from bacteria of different taxa or strains, landing on different surfaces, might adopt different configurations. For example, certain bacteria produce surfactant which can alter the morphology of a developing biofilm and allow them to expand over surfaces more efficiently [58, 59]. Our work suggests that such spreading phenotypes might be selected for in environments where there is little competition for resources, whereas a more compact clumping phenotype would have a selective advantage in an environment where competition for resources is high. In high competition environments, manifestation of this clumping phenotype would no doubt involve the production of cohesive polymers such as extracellular DNA, proteins, and polysaccharides.

Our work has been inspired by the observation that bacterial aggregates often form in the planktonic phase [11–13]. Aggregates are known also to form via the detachment of bacterial clumps from a mother biofilm [15, 60, 61]; should such aggregates land on a pristine surface, similar phenomena to those discussed here would be expected to arise. Moreover, our results could also be relevant to aggregates that form on the surface itself. In the classical picture of *P*. *aeruginosa* biofilm development, individual cells land on a surface, upon which they migrate and proliferate to form small aggregates (i.e. microcolonies). Surface-induced motility mechanisms [62, 63] such as twitching [64], crawling [65] and walking [66] have been implicated in this process. Once formed, such small aggregates would compete with surrounding cells for nutrients in much the same way as the pre-formed aggregates that we have investigated in this paper.

This study has focused on a single pre-formed aggregate seeding the surface and competing with initially unaggregated cells during biofilm formation. To further understand biofilms in nature, this work should be extended to investigate competition between multiple aggregates arranged on the surface, and competition between aggregates and mixed strains of bacteria, i.e., strains with different growth rates.

Recently cooperation within clumps of aggregated cells has been suggested to be a stepping stone in the evolution of multicellularity [26, 27]. Our study thus also hints that interesting social interactions might arise between cells within an aggregate. For all aggregate shapes, we observe heterogeneity in fitness among cells within the aggregate. This is particularly pronounced for the rounded aggregate, where cells at the top are strongly favoured while those in the centre of the aggregate hardly proliferate. Based on arguments recently put forward by West and Biernaskie [26, 27], one might predict that rounded aggregates would be favourable under conditions where cells within the aggregate are closely related, whereas spread aggregates, in which fitness differences between cells are less pronounced, might form where cells are less closely related. This leads to interesting further questions, e.g., when a rounded aggregate initiates biofilm growth, do the majority of cells in the aggregate “sacrifice” their future progeny in favour of their kin at the top? This idea supports previous suggestions that height plays a crucial role in competition within biofilms [33]. While previous work pointed to EPS production as a means to push progeny cells above the surrounding competitors [33, 67], our work shows that aggregate formation also provides a means to this end. Such a picture raises new questions about the evolutionary implications of bacterial aggregation.

## Supporting Information

S1 File (PDF)
