# Correction: Shaping the Growth Behaviour of Biofilms Initiated from Bacterial Aggregates

**DOI:** 10.1371/journal.pone.0154637

**Published:** 2016-04-27

**Authors:** The PLOS ONE Staff

The Data Availability Statement is incorrect. The correct statement should read as follows: Data are available from the Edinburgh DataShare repository (http://dx.doi.org/10.7488/ds/1340). The publisher apologizes for the error.
