# Supplementary material for: Shaping the Growth Behaviour of Biofilms Initiated from Bacterial Aggregates
Source: PLoS One. 2016 Mar 2;11(3):e0149683. doi: 10.1371/journal.pone.0149683 (PMC4774936; doi:10.1371/journal.pone.0149683)
Supplement: S1 File — (PDF) [file pone.0149683.s001.pdf]

Supplementary Material

Shaping the Growth Behaviour of Biofilms Initiated from Bacterial Aggregates

A Generating 2D bacterial aggregates

To generate bacterial aggregates of the appropriate shape, circular segments were extracted from simulation configurations of pre-grown biofilms as illustrated in Figures S1 and S2. Figure S1(a) shows a circle (red) with radius R and segmental region defined by the white area between the black horizontal line and the perimeter of the circle. The "surface-aggregate angle", θ, defines the angle between the black horizontal line (surface) and the tangent line of the circle at the point of contact with the surface.

The area, A, of a circular segment of radius R and with surface-aggregate angle of θ, is given by [1]

$$A = \frac{1}{2}(2\theta - \sin 2\theta)R^2. \tag{S1}$$

The corresponding arc length, s, of an aggregate defines the initial interface between the bacterial aggregate and the surrounding nutrient medium, and is computed according to

$$s = 2R\theta. \tag{S2}$$

To ensure our aggregates contained the same number of cells (∼100), the area in Equation S1 was fixed at 1257 μm^2.

The procedure for generating the starting aggregate configurations was performed as follows:

1. An *in silico* biofilm was grown for a simulation time of 7 days. This simulation was initiated with 200 cells randomly (uniform) distributed on the surface and a nutrient concentration of 5.4×10^{-2} g L^{-1} was used. A typical biofilm resulting from such a simulation was approximately 800 μm in height with some finger-like projections such as those in Figure S2.

2. A point, P, was defined in the middle of the biofilm (Figure S1 and S2). The point P is the reference point from which the origin, O, of a circle is computed. The point P was selected such that the density of cells within any segmental area was relatively uniform. Bacterial aggregates of shapes $\theta = 5, 10, 15, 20, 25, ..., 180^\circ$ with fixed area of 1257 μm^2 were then generated as in Figure S2.

3. For each θ, the radius of the corresponding circle was computed by solving Equation S1 for R. Using the radius, R, of this new circle, we computed the distance, X, to move the origin of this new circle above or below the point P according to

$$X = R\,\text{sin}\alpha, \tag{S3}$$

   where

$$\alpha = 90 - \theta. \tag{S4}$$

4. With the new origin and radius defined, we then extracted all bacteria within the radial distance from O. Note those bacteria located below the horizontal line (blue region) were excluded. The corresponding segment gave rise to a bacterial aggregate with a shape determined by the value of θ.

5. The vertical component of the point P was then subtracted from all bacterial coordinates so that the base of the aggregate was located on the surface as shown in Figure 1(a) of the main manuscript.

B Generating configurations of competing "single cells" in 2D

The width of the surface available to the surrounding competitor cells depends on the surface coverage of the aggregate. As surface coverage of the aggregate increases, $\theta \to 0^\circ$, the amount of available surface either side of the aggregate decreases. Surrounding cells were inserted at random positions

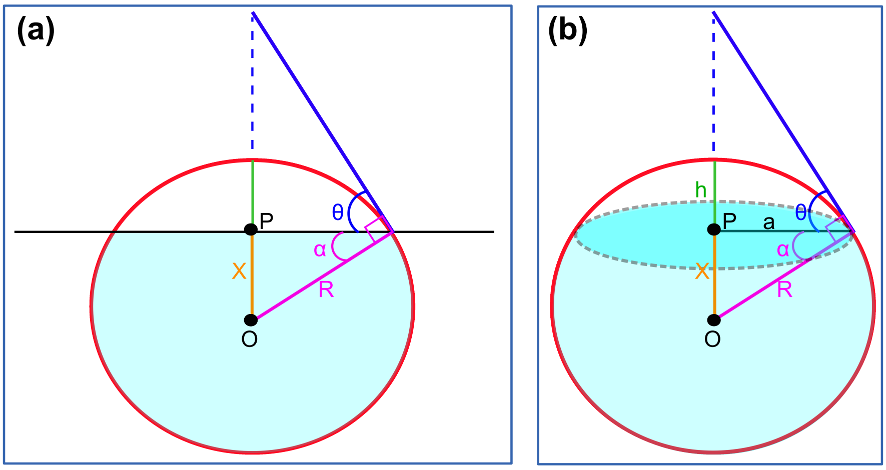

Supplementary Figure S1: Schematic representation of the geometry of aggregates in (a) 2D and (b) 3D. The light blue region represents the area not included when computing cells within a distance R from the origin, O. The angle θ is the contact angle of the aggregate with the surface.

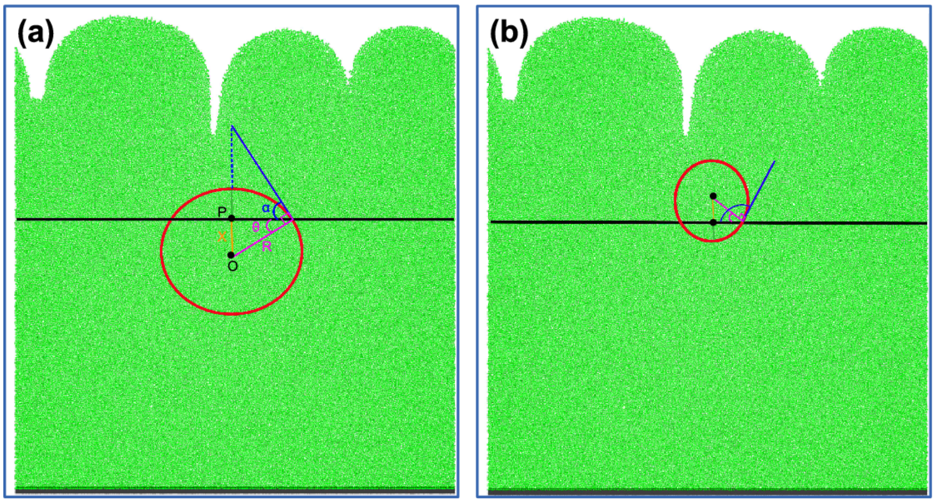

Supplementary Figure S2: Generating aggregates with different geometries but containing the same number of cells. Using pre-grown 2D biofilm configurations, green, aggregates of the desired shape can be generated using the geometric relationships from Figure S1(a). (a) Schematic showing how a spread aggregate is generated. (b) Schematic showing how a more rounded aggregate is generated.

uniformly distributed on the surface excluding the region occupied by the aggregate. To ensure a constant density of cells surrounding the aggregate, the number of cells in these regions were varied according to the width of surface available.

C Generating 3D aggregates

To generate aggregates in our 3D simulations, we performed an analogous process to that used for the 2D case. Configurations of spherical caps were transplanted from pre-grown biofilms using the geometrical relationships illustrated in Figure S1(b). The volume of a spherical cap is [2]

$$V_{cap} = \frac{1}{3}\pi R^3(2 - 3\sin\alpha + \sin^3\alpha), \tag{S5}$$

and the corresponding surface area of the cap is given by

$$S = 2\pi Rh, \tag{S6}$$

where the height, h, is given by

$$h = R - X \tag{S7}$$

A spherical cap volume, V_{cap}, of 5575 μm^3 was used for all values of θ, giving approximately 118 bacteria in each aggregate.

D Generating configurations of competing "single cells" in 3D

To surround the 3D aggregates with neighbours, we seeded the surface surrounding the aggregate with single cells. The number of these competing cells per square μm gave the density of cells on the surface. Cells that lay in the region defined by the radius of the spherical cap, a, were removed from this configuration. The aggregate configuration and the surrounding cell configuration were then combined to produce the initial state of the system.

E Multiple simulation runs in 2D and 3D

For our 2D simulations, four different aggregate configurations were generated for each value of θ, and for each of these configurations, 5 simulations of

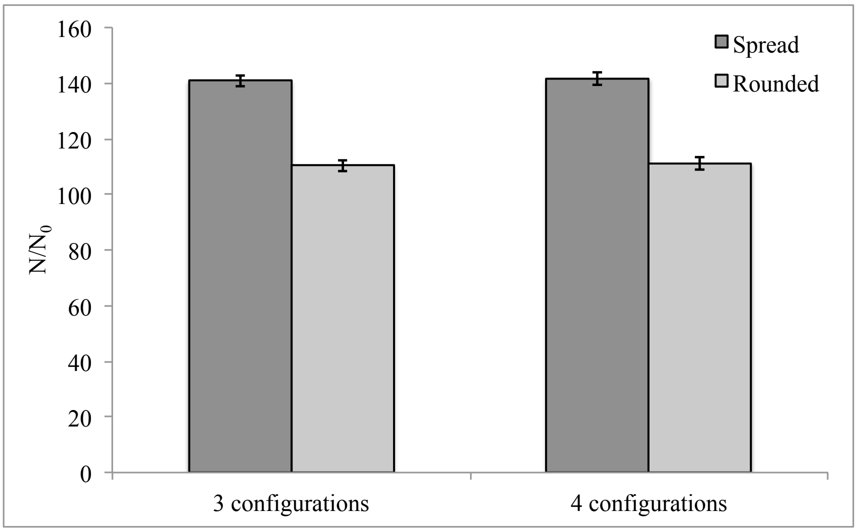

Supplementary Figure S3: Increasing number of simulations from 3 to 4 does not affect results. N/N_0 for the spread and rounded aggregate in the absence of surrounding cells on the surface. Error bars represent the standard deviation.

480 hours were performed using a different initial distribution of surrounding cells. This gave rise to a total of 20 simulations for each value of θ. Figure S3 shows that there is little difference in N/N_0 when going from three configurations (15 simulations in total) to four configurations (20 simulations in total) for the spread and rounded aggregates in the absence of surrounding cells on the surface (see main manuscript). From this control, we were satisfied that increasing the number of simulations by sampling more configurations would not change our results.

In 3D, only the spread aggregate, $\theta = 5°$, and the rounded aggregate, $\theta = 180°$, were simulated. Three different initial configurations were generated for each value of θ, and for each of these configurations 3 simulations of 72 hours were performed, each using different initial distributions of surrounding single cells. This gave rise to a total of 9 simulations for each value of θ.

Note that, in both the 2D and 3D simulations, the competitor cells on the surface and the aggregated cells are identical in their growth parameters (Table 1, main manuscript).

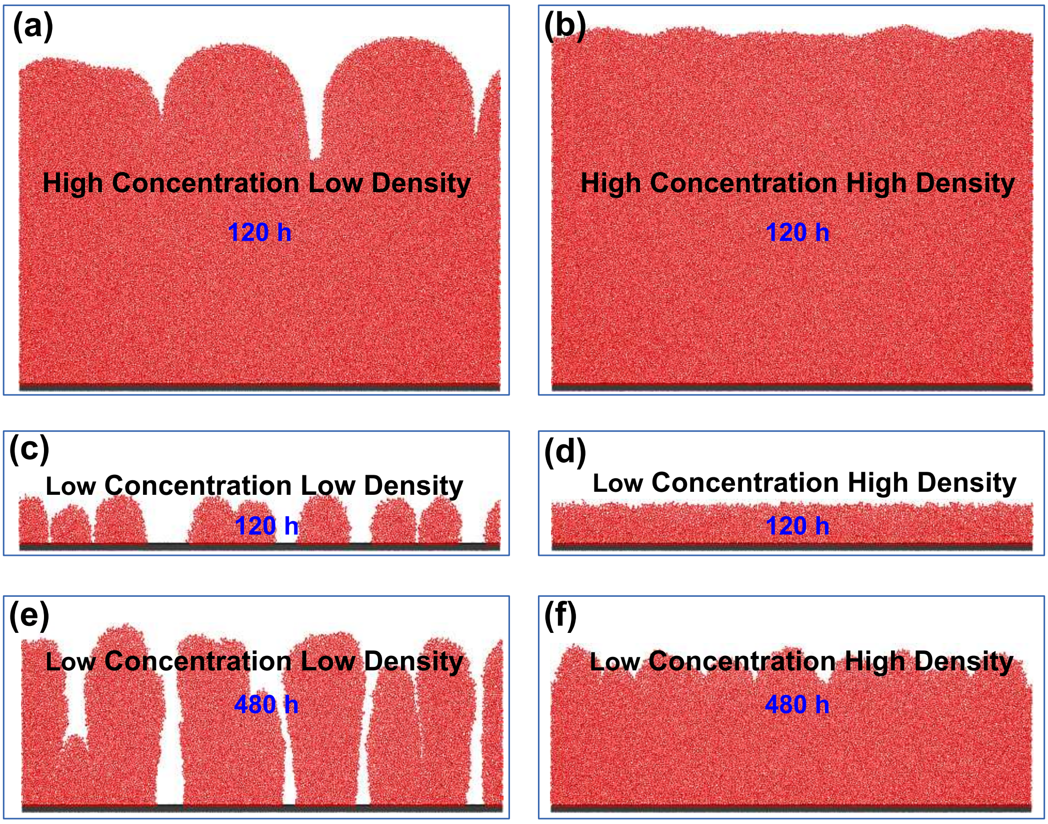

Supplementary Figure S4: Nutrient concentration and initial density of seeder cells affect biofilm morphology in the absence of initial aggregates: (a) Nutrient concentration = 5.4×10^{-2}g L^{-1}, Density of seeder cells = 0.01 cell μm^{-1}. (b) Nutrient concentration = 5.4×10^{-2}g L^{-1}, Density of seeder cells = 0.5 cell μm^{-1}. (c) Nutrient concentration = 5.4×10^{-3}g L^{-1}, Density of seeder cells = 0.01 cell μm^{-1}. (d) Nutrient concentration = 5.4×10^{-3}g L^{-1}, Density of seeder cells = 0.5 cell μm^{-1}. (e) Nutrient concentration = 5.4×10^{-3}g L^{-1}, Density of seeder cells = 0.01 cell μm^{-1}, 480 h. (f) Nutrient concentration = 5.4×10^{-3}g L^{-1}, Density of seeder cells = 0.5 cell μm^{-1}, 480h.

F Exploration of biofilm structure at low and high nutrient concentration without aggregates

To investigate the effect of nutrient concentration in general in our simulations, we simulated 2D biofilms in the absence of initial aggregates, at several

different nutrient concentrations and various densities of cells, which were initially positioned at random (uniform) locations across the surface. Figures S4 (a) and (b) show the resulting biofilms formed after 120 h at a nutrient concentration of 5.4×10^{-2}g L^{-1} for seeder cell densities of 0.01 cell μm^{-1} and 0.5 cell μm^{-1}. Clearly, the biofilms formed at this nutrient concentration are significantly thicker than those formed at lower nutrient concentration (Figures S4(c) to (f)). Comparing (a) and (b), we see that a low density of seeder cells leads to a less uniform biofilm morphology with finger-like projections.

After 120 h, at low nutrient concentrations (5.4×10^{-3}g L^{-1}), the biofilms remain very thin (Figures S4(c) and (d)). At this low concentration, the initial number of seeder cells has a marked effect on the final structure of the biofilm. A high density of seeder cells yields a biofilm with a uniform morphology whereas a low density of seeder cells yields a biofilm with a spatial structure that is clearly dependent on the initial configuration of the seeder cells. Figures S4(e) and (f) show that this effect persists at long times.

From Figures S4(a) and (e), it is clear that both longer times and/or higher nutrient concentration lead to larger biofilms. Selecting a bulk nutrient concentration and timescale to investigate the shape dependent fitness of aggregated clusters of bacteria is therefore non-trivial. In our simulations, we used the lower nutrient concentration of 5.4×10^{-3}g L^{-1} (see Table 1 in the main manuscript), comparable with previous biofilm simulations [3, 4, 5]. We also chose to run the simulations for 480 h in order to explore the long term growth dynamics, and to generate populations that were large enough to obtain good statistics. Timescales of 480 h have also been used in previous simulation studies [3, 4]. After 480 h growth using a bulk concentration of 5.4×10^{-3}g L^{-1}, our simulations produce biofilms that are approximately 200-300 μm in height (Figures S4(e) and (f)).

G Exploration of aggregate fate at different times

In the main manuscript, we computed N/N_0, as a function of the aggregate surface angle, θ, after 480 h. Here, we show that qualitatively similar results are obtained regardless of the time at which fitness is measured. Figures S5 shows N/N_0 as function of θ in the absence of competing cells (no red cells in Figure 1(a) of the main manuscript) at 4 different times. Evidently, the functional form of the curves does not change significantly with time.

In the highly competitive regime where we include competitor cells (red cells in Figure 1 of main manuscript) at a density of 0.5 cells μm^{-1}, the

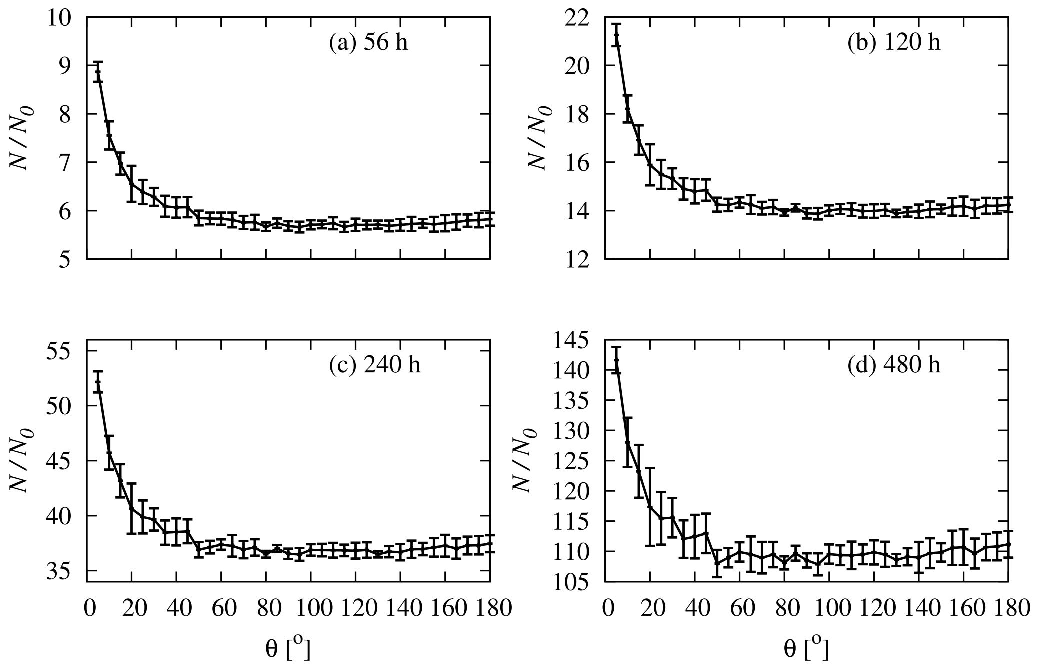

Supplementary Figure S5: Without competing cells on the surface, the functional form of the N/N_0 curves remains relatively constant over time. Note, however that the scale does change because more cells are produced as time increases: (a) 56 h. (b) 120 h. (c) 240 h. (d) 480 h. Error bars represent the standard deviation from 20 simulations.

situation is more complex. Here, the functional form of the curves changes during the course of the simulation (Figure S6). For early times, the curves are similar to the case without competition, suggesting that shape-dependent nutrient access is the still dominant factor in the early stages. As time increases, the rounded aggregates become increasingly "fitter" relative to the spread aggregates, until at 480 h, all aggregates with a surface aggregate angle greater than 75° are "fitter" than the spread aggregate. From this we can conclude that the initial height advantage of the rounded aggregate only becomes important at longer times.

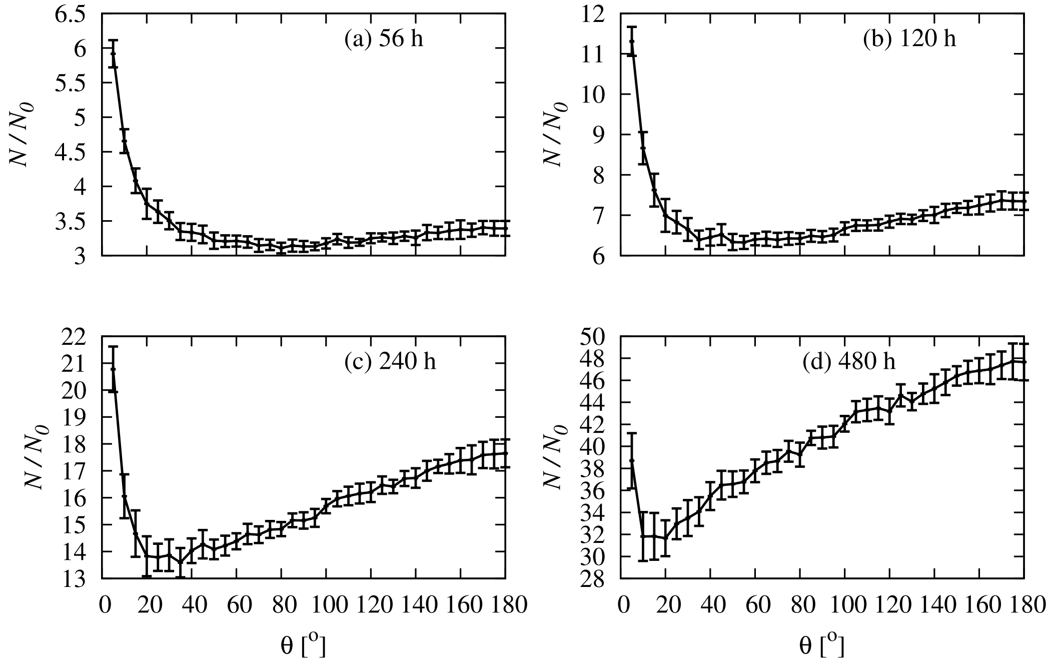

Supplementary Figure S6: With competing cells on the surface at a density of 0.5 cell μm^{-1}, the functional form of the N/N_0 curve changes over time: (a) 56 h. (b) 120 h. (c) 240 h. (d) 480 h. Error bars represent the standard deviation from 20 simulations.

H Difference in fate between spread and rounded aggregates at higher nutrient concentration

Here, we investigate the fate of the spread and round aggregate at a higher nutrient concentration, 1×10^{-2} g L^{-1}. Figure S7 shows that our conclusions remain valid at this nutrient concentration: at low density of seeder cells there is no difference in fate between the spread and rounded aggregates, whereas at higher density of initial neighbours, the rounded aggregate is favoured over its initially more spread counterpart. Note that these simulations were again run for 480 h.

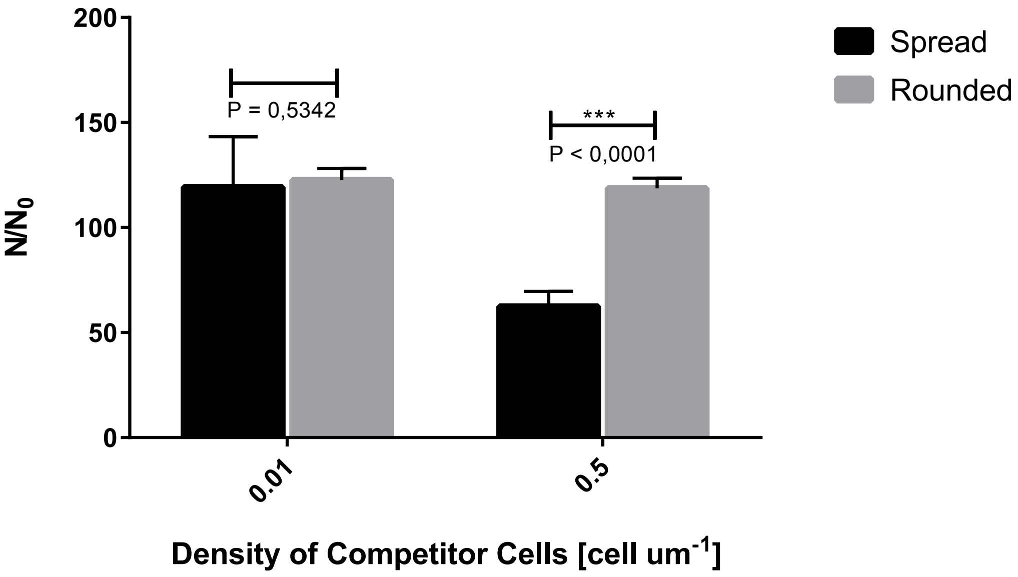

Supplementary Figure S7: At higher nutrient concentration, 1×10^{-2}g L^{-1}, rounded aggregates are more favourable for growth. At low density of seeder cells (0.01 cell μm^{-1}) there is no significant difference in N/N_0 between the spread and rounded aggregates. At high density (0.5 cell μm^{-1}), the rounded aggregate again produces more progeny per initial cells relative to that of the spread. Note that these data were generated from simulations representing 480 h of biofilm growth.

I The effect of aggregate shape on the growth of the active layer

In the main manuscript we discussed cell growth rate heterogeneity within the biofilm and the presence of a well-defined active layer in the growing aggregate. The depth of the active later has been shown to be very important in determining biofilm structure and the degree of segregation amongst cell groups within the community [6]. It has also been discussed in extensive mathematical detail by Dockery and Klapper [7]. Here we look at the growth of the active layer.

In the analysis that follows, we simply define the active layer as the region of the growing aggregate in which all cells grow with a growth rate greater than 1×10^{-15}h^{-1} (see Figure 6 of main manuscript), and we assess the

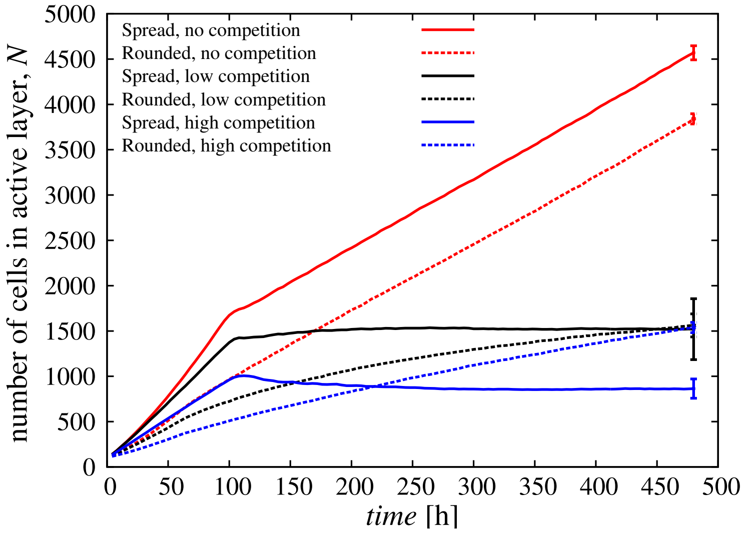

Supplementary Figure S8: Dynamics of the growing layer determine aggregate growth behaviour. The number of growing cells in the spread and rounded aggregates at various competitive regimes. For clarity the error bars, representing the standard deviations, are only shown for the final data points. The standard deviations at these points are maximal.

number of cells in this layer. This choice is arbitrary; what is important is the qualitative difference in behaviour between the aggregate shapes.

Figure S8(a) shows the number of active cells within the spread ($\theta = 5°$) and rounded ($\theta = 180°$) aggregates at varying degrees of competition; dictated by the density of surrounding unaggregated cells on the surface. In the absence of competition, the number of active cells within the spread and rounded aggregates increases with time. Initially the number of active cells in the spread aggregate grows faster than that for its rounder counterpart, however for $t > 100$ h, the number of active cells in both aggregates increases with similar rates (slope of red curves). This linear increase determines the super-linear increase in the corresponding aggregate populations in Figure 3 of the main manuscript: a linear increase in the number of active cells translates to a super-linear increase in the total aggregate population.

In all competitive regimes, the number of cells in the growing layer of the spread aggregates becomes constant after $\sim$100 h. A constant population of active cells in the growing layer translates to linear growth behaviour of the total aggregate population (compare solid blue curve with the dashed red curve in Figure 3 of the main manuscript). For the rounded aggregates, this transition to the constant regime does not occur within the timescales of our

simulations, and the layer continues to grow monotonically.

Comparing the spread and rounded aggregates (solid curves with dashed curves), with competition, we see that growth of the active layer always increases faster for the spread aggregate before levelling off after 100 h. However, later in the simulation, the number of active cells in the rounded aggregates becomes greater. The time at which this crossover occurs increases with increasing levels of competition. The increasing growth of the active layer in the rounded aggregate at high competition, $\rho = 0.5$ cell μm^{-1}, explains why its number of progeny becomes larger than that of the spread aggregate in Figure 3 (main manuscript), and why its structures tend to fan outwards at the top (Figure 4(d)).

J Aggregate simulations in 3D

The results presented in the main manuscript are for 2D simulations, for reasons of computational feasibility. To validate our results further, we also ran some simulations in 3D. Figure S9 shows simulation snapshots after 72 h for the spread and rounded aggregates in the presence of low and high densities of competitor cells. As in our 2D simulations (Figures 4(b) and (d) of the main manuscript), the rounded aggregate persists as a dominant structural feature at both low and high densities of competitor cells. The spread aggregate on the other hand becomes swamped by the surrounding competitors and after 72 h is structurally indistinguishable from the rest of the biofilm (as in our 2D simulations, Figures 4(b) and (d) of the main manuscript).

N/N_0 for the spread and rounded aggregates is plotted in Figure S10 and is analogous to the data shown in Figures 8 of the main manuscript for the 2D simulations. As in the 2D case, the number of progeny per initial cell of the aggregates decreases with increasing density of surrounding cells. In the absence of competition, the spread aggregate again produces significantly more progeny than the rounded aggregate, however as the competition on the surface is increased, the number of progeny produced by cells in the rounded aggregate increases relative to that of the spread.

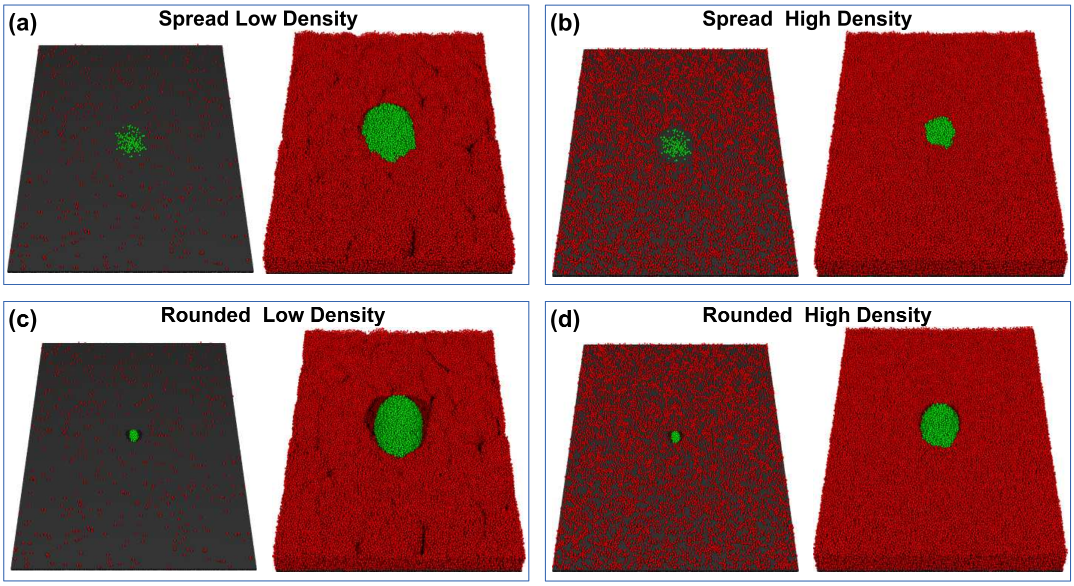

Supplementary Figure S9: 3D simulation snapshots showing initial and final configurations of the spread and rounded aggregates at low and high density of surrounding cells. (a) Spread aggregate, Density of seeder cells = 0.002 cell μm^{-2}. (b) Spread aggregate, Density of seeder cells = 0.03 cell μm^{-2}. (c) Rounded aggregate, Density of seeder cells = 0.002 cell μm^{-2}. (d) Rounded aggregate, Density of seeder cells = 0.03 cell μm^{-2}.

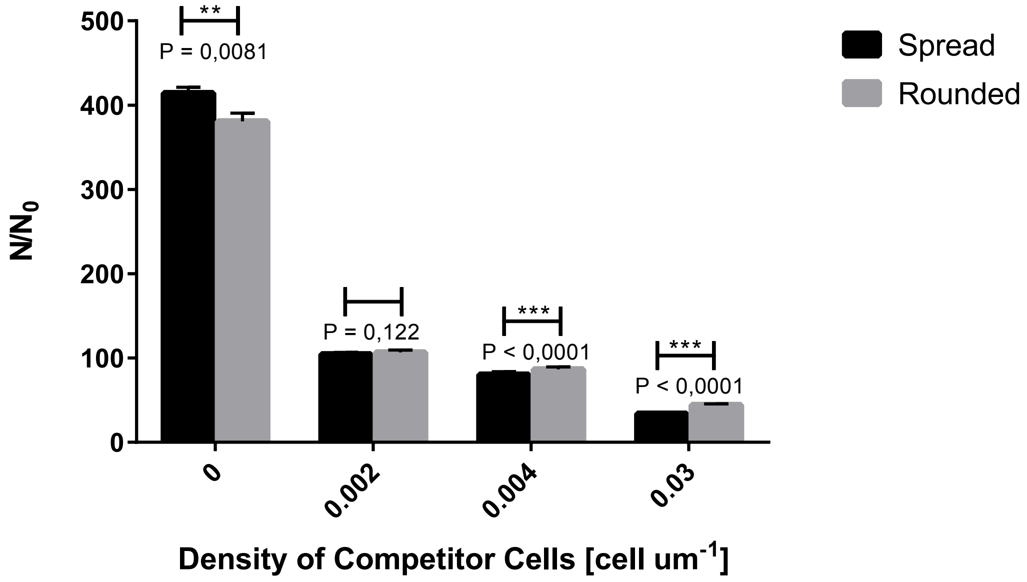

Supplementary Figure S10: N/N_0 of the 3D rounded aggregate with respect to the 3D spread aggregate increases with the density of neighbouring cells on the surface. In 3D, the trend is similar to that observed in 2D. The spread aggregate is significantly favoured when there is no competition on the surface however with increasing density the rounded aggregate is more favourable for growth.

References

[1] Weisstein, E. W. “Circular Segment”. From MathWorld–A Wolfram Web Resource. Available at: http://mathworld.wolfram.com/CircularSegment.html

[2] Weisstein E. W. “Spherical Cap”. From MathWorld–A Wolfram Web Resource. Available at: http://mathworld.wolfram.com/SphericalCap.html

[3] Xavier, J. B., Picioreanu, C., Rani, S. A., van Loosdrecht, M. C. M. , Stewart, P. S., Biofilm-control strategies based on enzymic disruption of the extracellular polymeric substance matrix–a modelling study, *Microbiol.* **151**, 3817-3832 (2005).

[4] Xavier, J. B., Foster, K. R., Cooperation and conflict in microbial biofilms. *Proc. Natl. Acad. Sci. USA* **104**, 876-881 (2007).

[5] Lardon, L. A., Merkey, B. V., Martins, S., Dtsch, A., Picioreanu. C., Kreft, J.-U., & Smets, B.F., iDynoMiCS: Next-Generation Individual-Based Modelling of Biofilms. *Environ. Microbiol.* **13**, 2416-2434 (2011)

[6] Nadell, C. D., Foster, K. R., & Xavier, J.B., Emergence of spatial structure in cell groups and the evolution of cooperation. *PLoS Comput. Biol.* **6**, e1000716 (2010)

[7] Dockery, J., & Klapper, I., Finger formation in biofilm layers. *SIAM J. Appl. Math* **62**, 853-869 (2001).
